# Targeting miRNA by Natural Products: A Novel Therapeutic Approach for Nonalcoholic Fatty Liver

**DOI:** 10.1155/2021/6641031

**Published:** 2021-08-13

**Authors:** Mehdi Zobeiri, Fatemeh Parvizi, Mohammad Reza Kalhori, Mohammad Bagher Majnooni, Mohammad Hosein Farzaei, Mohammad Abdollahi

**Affiliations:** ^1^Internal Medicine Department, Imam Reza Hospital, Kermanshah University of Medical Sciences, Kermanshah 6734667149, Iran; ^2^Pharmaceutical Sciences Research Center, Health Institute, Kermanshah University of Medical Sciences, Kermanshah 6734667149, Iran; ^3^Regenerative Medicine Research Center, Kermanshah University of Medical Sciences, Kermanshah 6734667149, Iran; ^4^Toxicology and Diseases Group, Pharmaceutical Sciences Research Center (PSRC), The Institute of Pharmaceutical Sciences (TIPS) and Department of Toxicology and Pharmacology, School of Pharmacy, Tehran University of Medical Sciences, Tehran 1417614411, Iran

## Abstract

The increasing prevalence of nonalcoholic fatty liver disease (NAFLD) as multifactorial chronic liver disease and the lack of a specific treatment have begun a new era in its treatment using gene expression changes and microRNAs. This study aimed to investigate the potential therapeutic effects of natural compounds in NAFLD by regulating miRNA expression. MicroRNAs play essential roles in regulating the cell's biological processes, such as apoptosis, migration, lipid metabolism, insulin resistance, and adipocyte differentiation, by controlling the posttranscriptional gene expression level. The impact of current NAFLD pharmacological management, including drug and biological therapies, is uncertain. In this context, various dietary fruits or medicinal herbal sources have received worldwide attention versus NAFLD development. Natural ingredients such as berberine, lychee pulp, grape seed, and rosemary possess protective and therapeutic effects against NAFLD by modifying the gene's expression and noncoding RNAs, especially miRNAs.

## 1. Introduction

Nonalcoholic fatty liver disease (NAFLD) is one of the chronic liver diseases nowadays which threatens human health leading to liver dysfunction. NAFLD is caused by the abnormal accumulation of fat (more than 5% of liver weight), especially triglycerides, in the liver of people who are not addicted to alcohol [1, 2]. As this disease's incidence has a close association with lifestyle, it is more common in developed countries. In the absence of proper treatment, this disease can progress, and nonalcoholic steatohepatitis (NASH) could be created [3]. NASH also can increase the risk of cirrhosis and hepatocellular carcinoma (HCC) due to environmental conditions and genetic factors [4].

NAFLD is a multifactorial disease that initiates and develops due to the interactions between various risk factors. In addition to diet, hyperlipidemia, diabetes mellitus, environmental factors, obesity, insulin resistance (IR), and genetic factors such as gene expression or single-nucleotide polymorphism (SNP) have a crucial role in the formation of NAFLD [1, 5]. The environmental risk factors include air pollution, water or food pollution, and chemical materials. The most significant air pollution is particles less than 2.5 micrometres that are harmful to human health and are involved in the pathogenesis of NAFLD through nuclear factor-*κ*B (*NF-κB*), c-Jun *N*-terminal kinase- (*JNK*-) activator protein 1 (*AP1*), and toll-like receptor 4 (*TLR4*) activation [5, 6]. Furthermore, the principal food or water pollution and chemical risk factors that include aflatoxin, trichloroethylene, pesticides, heavy metals, and trihalomethanes also have a significant impact [5, 7].

Moreover, many genetic and epigenetic risk factors performed crucial roles in the susceptibility and progression of NAFLD. Recent studies have shown that a wide variety of modifications include copy number variation, structural variation, genes, long noncoding RNAs (lncRNAs), or microRNA expression changes in NAFLD [8]. Some of these genes (lipid biosynthetic regulating transcription factors, nuclear receptors, fibrogenesis, and inflammatory response factors) are involved in NAFLD genetic susceptibility [9], for instance, the NLR family pyrin domain-containing 6 (*NLRP6*), NLR family pyrin domain-containing 3 (*NLRP3*), and interleukin-18 (*IL*-*18*) genes' expression or SNPs in patatin-like phospholipase domain-containing protein 3 (*PNPLA3*-rs738409 and rs6006460) [2, 10]. Transmembrane 6 superfamily member 2 (TM6SF2-rs58542926) and membrane-bound O-acyltransferase domain-containing 7 (*MBOAT7*-rs641738) genes play essential roles in the initiation or progression of this disease [11]. Noncoding RNAs (ncRNAs) such as microRNAs (miRNAs) and LncRNA could not encode any protein and transcribe from the cell genome. MicroRNAs play essential roles in regulating the cell's biological processes, such as apoptosis, migration, lipid metabolism, insulin resistance, and adipocyte differentiation by downregulating its targeting genes [12, 13]. According to this, gene structure changes (epigenetic modifications such as methylation and acetylation) or gene expression changes are among the NAFLD causes. Therefore, miRNAs' role in the biogenesis, pathogenesis, development, and progression of this disease is not unusual [14, 15].

Natural products (NPs) are chemical agents derived from living organisms such as bacteria, fungi, plants, and animals. Research has shown that some of these substances have therapeutic effects and could be used as pharmacological agents to treat various diseases [16]. Nowadays, more than 80% of medicines are made of natural compounds [17]. Subsequently, with the development of molecular techniques, it has been found that one of the beneficial effects of these natural products on the disease is through modifying the expression of the gene and noncoding RNAs, especially miRNAs. These substances produce epigenetic changes in the cell by modification of histones and DNA. As a result, an increase or decrease in the expression of some genes or miRNAs occurs [18]. Thus, using natural products could adjust miRNA profiles, inhibit metastasis, increase drug susceptibility, inhibit cancer progression, and treat or prevent many diseases such as NAFLD [19]. As identified, some of these compounds, such as ellagitannin, resveratrol, curcumin, genistein, and epigallocatechin-3-gallate, could inhibit proliferation, induce apoptosis, and modify cell behaviour through the effect on miRNAs' expression profiles [20].

The aim of this study is to investigate the usefulness of natural agents (as an essential factor in creating epigenetic changes) in the regulation of miRNA for the treatment of nonalcoholic fatty liver disease. We expect that this would provide new plans in NAFLD therapies by using natural products.

### 1.1. A Brief Overview of miRNAs

Noncoding RNAs (ncRNAs) are groups of RNAs that do not synthesize any proteins. In general, according to their length, ncRNAs are divided into two groups. Small ncRNAs are less than 200 nucleotides, and long ncRNAs consist of more than 200 nucleotides. Small ncRNAs consist of small nuclear RNAs (snRNAs), PIWI-interacting RNAs (piRNAs), transfer RNAs (tRNAs), and miRNAs. Among ncRNAs, microRNAs (miRNAs) are the most studied molecules to date [21]. miRNAs are highly conserved small noncoding RNAs involved in regulating the expression of approximately 60% of mammalian genes at the posttranslation level by multiple mechanisms [22]. These molecules are located on the intergenic, exonic, or intronic regions of all human chromosomes, except for the Y chromosome [20].

Lin-4 was the first miRNA discovered by Victor Ambros in 1993 that targeted the 3′ UTR of lin-14 mRNA from *Caenorhabditis elegans*. After many studies and the advancement of human knowledge, the number of known molecules has increased dramatically. Today, over 48860 mature microRNAs and 38589 hairpin precursors from 271 organisms are included in the miRBase database [23, 24]. In vitro and bioinformatics studies have shown that many miRNAs can target three prime untranslated regions (3′ UTR) of a single gene. In contrast, miRNA solely can bind to the 3′ UTR of several genes and regulate their expression [25].

Moreover, based on their function, miRNAs are divided into two groups. The first group is oncomiR, which is overexpressed in diseases or cancers and inhibits genes that are essential for human health. The second group is tumour suppressor miRNAs that are downregulated or silenced in cancers and illnesses. As a result, their inhibitory trait is removed from the oncogenic signalling pathways [18]. miRNAs are affected by genetic and epigenetic modifications occurring in the cell genome. For instance, any agent that leads to DNA methylation of the loci of miRNAs could reduce miRNA expression; in contrast, the region's demethylation increases miRNA expression [26]. Therefore, by modifying the methylation or demethylation of genes responsible for the generation or improvement of NAFLD through various materials or methods, such as natural products, we can catch a basic level to manage this illness [24].

miRNAs could bind to mRNA 3′ UTR through their seed region. The seed position is in the 5′ untranslated region (5′ UTR) of miRNAs between nucleotides 2 and 8. Since this region is not more than a few nucleotides in length, its complementary sequence may be present in several different mRNAs so that miRNA may target several other genes [27]. It has also been shown that miRNA may have more than one complementary site on the 3′ UTR of its target gene. Therefore, miRNA's effect on the gene is more significant than miRNA by only one complementary binding site [28]. Although these single-strand RNAs (18–28 nucleotides) (miRNAs) have an essential role in regulating and controlling the biological activities of healthy cells, they are also involved in the development and pathogenesis of various diseases, especially cancer. To date, there have been numerous studies showing that miRNAs are involved in proliferation, differentiation, apoptosis, and cell migration [19, 29].

The miRNAs' biogenesis is initiated at the nucleus by RNA polymerase II. First, miRNAs are transcribed as primary miRNA (pri-miRNA) by several thousand nucleotides. Then, pri-miRNA is cleaved by Drosha and DGCR8 to produce pre-miRNA (70–100 nt) [30]. miRNA is then transported from the nucleus to the cytoplasm by RanGTP and exportin 5. Dicer binds to it in the cytoplasm, performs secondary cleavage, and produces double-stranded RNA (mature miRNA) [31]. After mature miRNA is inserted into the RNA-induced silencing complex (RISC), it could target 3′ UTR mRNAs of the target genes [32].

Nevertheless, some reports indicated that miRNAs could bind to 5′ UTR of the target gene in addition to the 3′ UTR [15]. For example, hsa-miR-24-3p, miR-34a, miR-US25-1, and miR-103a-3p could target 5′ UTR of the c-Jun activation domain-binding protein 1 (Jab1), axis inhibition protein 2 (AXIN2), human cytomegalovirus (HCMV), and GPRC5A, respectively [33, 34]. miRNAs can increase the stability or expression of target genes by binding to 5′ UTR of theirs. For example, miR-10a or miR-122 increases the expression and strength of ribosomal protein mRNAs and RNA of the hepatitis C virus, respectively [35, 36].

miRNAs could regulate gene expression in both direct and indirect methods. Instantly, they destroy or inhibit the translation of target mRNA. Nevertheless, indirectly, they could inhibit upstream genes that are the inhibitor, activator, or transcription factor for the target gene [37, 38]. Alterations of miRNAs have been reported in a wide range of diseases, including human pathological liver conditions. Previous studies showed that these molecules have an essential role in NAFLD's pathogenesis and could apply as a potential therapeutic target [4].

### 1.2. MicroRNAs as a Therapeutic Target for NAFLD

Liver biopsy is the standard method for the diagnosis of NAFLD. However, this method is an invasive procedure that can cause many problems and discomfort for patients, especially children and the elderly. Therefore, attaining a new and noninvasive method such as serum biomarkers (may be miRNAs) and ultrasound and imaging techniques is essential to detect it before clinical symptoms occur [39]. By regulating the expression or activity of many genes, miRNAs play a crucial role in regulating lipid metabolism in the liver [40]. There is a close relationship between NAFLD and the expression profile of miRNAs. It has been reported frequently that these molecules' expression changes in animal models and NAFLD/NASH patients.

Furthermore, miRNAs can simultaneously affect several genes from different molecular and signalling pathways (glucose metabolism, lipid metabolism, inflammation, and oxidative stress). This finding shows the significance of miRNAs as therapeutic target biomarkers [24, 41, 42]. In addition to body tissues, miRNAs are significantly present in body fluids such as serum, saliva, plasma, and urine to be used for early detection, prognosis, or treatment monitoring of diseases [43].

Circulating miRNAs or exosomal miRNAs are resistant to RNAse degradation due to their complexation with lipids, proteins, or lipoproteins. These molecules are found in the body fluids due to cellular damage or cell secretion and are detectable by available methods [44]. Exosomal miRNAs or exomiRs play fundamental functions in cellular communications by the entrance to the intercellular space. They are sometimes tissue-specific, and their expression changes in pathological conditions. Therefore, they have this potential to be employed as a molecular marker for NAFLD [45, 46].

Many studies have been performed to determine the relationship of circulating miRNAs with NAFLD as a novel potential biomarker. In this regard, miRNAs which are most important include miR-99a-3p, miR144-3p, miR-200b-5p, miR-200-3p [47], miR-34a, miR-122, miR-16 [48], miR-21, and miR-451 [49]. Some of these miRNAs, such as miR-34a-5p, miR-375, and miR-301a-3p, could also indicate severity from NAFLD [50].

Moreover, in one study, 84 circulating miRNAs were analyzed to determine which of them are associated with NAFLD. The results showed that some miRNAs (miR-192, miR-122, miR-19a/b, miR-375, and miR-125b) are overexpressed in the steatosis sample. It further supposed a strange relationship between miRNA-192, miR-122, and miR-375 and nonalcoholic steatohepatitis (NASH) [51]. It has been shown that improper nutrition can lead to obesity by altering 6% miRNAs [52]. Here, we discuss if particular miRNAs are correlated by the progression of NAFLD in humans.

### 1.3. miR-34a

One of the miRNAs that increased in high-fat-fed mice is miR-34a. Human studies have likewise shown that this miRNA is closely associated with metabolic syndrome and NASH severity. Moreover, its expression in the tissue and serum of NAFLD/NASH patients increased [4, 53]. The role of miR-34a in NAFLD's pathogenesis is due to the development of lipid accumulation in hepatocytes and decreasing fatty acid B-oxidation through inhibition of the Sirtuin 1 (*SIRT1*) gene [54]. miR-34a could indirectly reduce peroxisome proliferator-activated receptor alpha (*PPAR*-*α*) gene activity and also could enhance sterol regulatory element-binding transcription factor 1c (*SREBP*-*1c*), peroxisome proliferator-activated receptor-*γ* coactivator-1*α* (*PGC-1α*), and farnesoid *X* receptor (*FXR*) gene activity. Thus, by impairing metabolic sensors, NAFLD-associated lipids' metabolism will be deregulated [55, 56]. Additionally, restraint of the *SIRT1* gene expression could activate proapoptotic genes such as *P53* and Src homology 2 domain-containing- (SHC-) transforming protein 1 isoform p66Shc variant (*P66SCH*), thereby increasing the susceptibility of hepatocyte cells to apoptosis and oxidative stress [57, 58]. Therefore, downregulation of miR-34a leads to the rational expression of the 3-hydroxy-3-methylglutaryl-coenzyme A reductase (*HMGCR*), *PPAR-α*, and *SIRT1* genes and improves the steatosis [56].

### 1.4. miR-33a/b

Another miRNA that plays an essential role in fatty liver disease by influencing lipid metabolism is miR-33. In humans, this miRNA has two members (miR-33a and miR-33b) that are located in the intron region of the sterol regulatory element-binding transcription factor 2 (*SREBP2*) and sterol regulatory element-binding transcription factor 1 (*SREBP1*) genes, respectively. These two genes play fundamental roles in controlling cholesterol and lipid synthesis [59, 60]. Moreover, these miRNAs play a vital role in regulating insulin signalling, de novo lipogenesis, triglyceride accumulation, and fatty acid oxidation. Consequently, inhibition of their expression or function could increase fatty acid oxidation, insulin sensitivity, elevated serum HDL, and decreased serum VLDL [61, 62]. Sirtuin 6 (*SIRT6*) and insulin receptor substrate 2 (*IRS-2*) are the target genes of miR-33, which play an essential role in controlling glucose metabolism. In vivo and in vitro studies suggest that overexpression of miR-33 plays a critical role in NAFLD development by affecting lipids and carbohydrate metabolism [41, 60, 61].

### 1.5. miR-155

One of the miRNAs that was downregulated in the serum and liver tissue of NAFLD patients is miR-155. Overexpression of miR-155 by any instruments led to a decrease in the expression of lipid metabolism-related genes such as *SREBP1*, liver *X* receptor (*LXR*), and fetal alcohol syndrome (*FAS*), thereby reducing intracellular lipid accumulation [63, 64]. Overall, the downregulation of the miR-155 expression level, with its crucial role in expanding adipose tissue mass, could facilitate NAFLD and obesity.

### 1.6. miR-451

Many studies have shown that the expression level of miR-451 decreased in high-fat-fed mice (HFD), hepatocyte- (HepG2-) treated cells with palmitic acid, and patients with NASH symptoms. As miR-451 is a negative regulator for proinflammatory cytokines (such as tumour necrosis factor-*α* (*TNF-α*), interleukin-8 (*IL-8*), and *NF-κB p65* subunit), its downregulation enhances the excretion of these cytokines through the AMP-activated protein kinase (AMPK)/AKT pathway [65]. Therefore, another proper objective for NAFLD treatment is the upregulation of miR-451 by natural products. The downregulation of the miR-451 level could facilitate NAFLD and obesity due to its significant role in expanding adipose tissue mass.

### 1.7. miR-375

Another miRNA that is considered for molecular targeting in the treatment of NAFLD is miR-375. This miRNA expression increased in the liver and serum of NAFLD patients and high-fat-fed mice, respectively, compared with steatosis samples and the control group. MiR-375 is a crucial regulator of glucose homeostasis, so its downregulation leads to a reduction in the amount of adiponectin receptor 2 (*AdipoR2*), interleukin-6 (*IL-6*), leptin, and *TNF-α*, ultimately reducing the lipid accumulation [51, 66].

### 1.8. miR-192

The miR-192 expression is altered in fatty liver disease. This miRNA has a profibrogenic power and decreases NASH. Nevertheless, its serum expression increased due to hepatocytes' secretion during the NASH's pathophysiological stages [67, 68].

### 1.9. miR-27a/b

miR-27a/b is one of the molecules that plays an essential role in abiogenesis. Its overexpression inhibits *SREBP1* and *FAS* genes and increases lipolysis, secretion of free fatty acids, and glycerol from the cell [69, 70].

### 1.10. miR-24

Another miRNA that increased in animal or human models of NAFLD is miR-24. As previously shown, miR-24 overexpressed in the HepG2 cell line was treated by fatty acids and in the liver of high-fat-fed mice. This miRNA could increase lipid accumulation in the hepatocytes by targeting the insulin-induced gene 1 (*Insig1*) gene that is an inhibitor of lipogenesis [53, 71]. Therefore, miR-24 inhibitors could play a crucial role in improving NAFLD.

### 1.11. miR-149

Studies have shown that there is a close relationship between NAFLD and miR-149 expression. For example, the expression of this miRNA in NAFLD mice and fatty acid-treated HepG2 cell lines increased. miR-149 leads to increased lipid accumulation and lipogenesis by inhibiting the fibroblast growth factor 21 (*FGF-21*) gene. Consequently, the downregulation of miR-149 and upregulation of *FGF-21* by pharmacological methods can enhance lipid metabolism and could improve NAFLD [72, 73].

### 1.12. miR-21

The expression of miR-21 in NAFLD patients' serum and liver tissue is different from that of healthy controls. This miRNA expression is increased in patients' hepatocytes, whereas serum expression is lower than in the control group [49, 67]. Nutrition plays a vital role in miRNA expression; previous studies have shown that miR-21 increased in the liver of high-fat mice and fatty acid-treated HepG2 cells. miR-21 affects lipogenesis, NAFLD, and cancer induction by its inhibitory effect on human polybromo-1 (*HPB1*) and *PPAR-α* [74, 75].

### 1.13. miR-122

miR-122 is the most abundant hepatic miRNA. It considers for approximately 70% of miRNAs expressed in that tissue and plays a fundamental role in the maturation, differentiation, and proliferation of hepatocytes [76, 77]. Serum level of miR-122 is associated with liver fibrosis in NAFLD patients, and its accuracy in describing NAFLD severity is higher than liver enzymes such as alanine aminotransferase (ALT) and aspartate aminotransferase (AST) [67]. Another notable feature of this biomarker is that its expression in NASH samples is approximately 7.2 and 3.1 fold, respectively, compared to healthy and steatosis samples [51]. However, there is a difference between serum and liver expression of miR-122 in NAFLD individuals. In other words, miR-122 tissue expression decreased in NAFLD/NASH patients compared to controls, but in contrast, its serum expression was upregulated in NAFLD/NASH patients [4, 78]. The increased serum level is that hepatocytes' destruction releases it in the fluid between the cells and blood [79]. Therefore, it can be used as a biomarker for noninvasive diagnosis of fibrosis development and liver injury. Studies revealed that mice lacking expression of this miRNA showed an increase in lipogenesis, lower serum cholesterol/triglyceride levels, fibrosis enhancement, NASH, and hepatocellular carcinoma, in addition to increased expression of TNF‐*α*, IL-6, and C-C motif chemokine ligand 2 (CCL2) genes [80, 81]. Further investigations showed that miR-122 increased the expression of acetyl-CoA carboxylase (*ACC1*), diacylglycerol O-acyltransferase 2 (*DGAT2*), *FAS*, and *SREBP1* genes that play essential roles in de novo lipogenesis [40, 76, 82].

### 1.14. miR-185

miR-185 is another miRNA involved in regulating the insulin signalling pathway, cholesterol homeostasis, and lipid metabolism that is downregulated in fatty acid-treated HepG2 cells (palmitic acid). Furthermore, overexpression of miR-185 in the C57BL/6 mouse model of NAFLD (high-fat diet mice) has been shown to decrease liver steatosis and increase insulin sensitivity. Molecular studies have also demonstrated that miR-185 performs its function by inhibiting the expression of lipid metabolism-related genes, including the *SREBP1c*, *HMGCR*, *FAS*, and *SREBP2* genes, and induces insulin sensitivity by promoting the phosphatidylinositol-3-OH kinase (PI3K)/AKT2 pathway via enhancing IRS-2 gene expression [83]. Therefore, it is one of the potential therapeutic targets in the NAFLD and could be overexpressed using dendrosomal curcumin [25].

Due to miRNAs' function in regulating different biological pathways, these molecules are considered therapeutic targets nowadays. Since these miRNAs could regulate the gene expression, up-/downregulation is a beneficial mechanism for preventing or treating various diseases [84]. The activity and function of miRNAs can be modified using competing endogenous RNAs (ceRNAs) such as lncRNAs and circular RNAs (circRNAs). Numerous studies have shown that one or more miRNAs' expression can be altered using natural products [20].

### 1.15. Is Natural Product Clinically Useful in NAFLD?

NAFLD's current pharmacological management, including drug and biological therapies, is expensive, possesses temporary relief, and has some adverse effects. Numerous clinical studies confirmed the ability of natural products in the management of NAFLD. In this context, various dietary fruits or medicinal herbal sources have received worldwide attention versus NAFLD development. This section represents the advantages of natural products in the management of NAFLD in human studies.

In a randomized, single-blind clinical trial, Shidfar et al. investigated the effects of extra virgin olive oil on the severity of steatosis in NAFLD patients on a weight loss diet. Fifty patients (19 women and 31 men) with nonalcoholic fatty liver were randomized to receive the hypocaloric diet enriched with olive oil (olive oil group) or the hypocaloric diet with normal fat (control group) for 12 weeks. It was found that ALT and AST levels significantly decreased in the olive oil group than in the control group. The result showed that consuming a diet containing olive oil enhances weight loss's beneficial effects and improves liver enzymes' level [85].

Because curcumin significantly improves lipid-modifying hepatic steatosis, a clinical study was reviewed to evaluate its efficacy in patients with NAFLD. In a randomized, double-blind, placebo-controlled trial, Rahmani et al. studied the effect of curcumin on the liver fat content on forty patients in the curcumin group (70 mg) and forty patients in the placebo group for eight weeks. Curcumin showed a significant decrease in serum levels of total cholesterol, liver fat content, triglycerides, body mass index, aspartate aminotransferase, and alanine aminotransferase [86]. In another work, Panahi et al. investigated the effects of curcumin on the metabolic profile in 87 patients with grades 1–3 of NAFLD. After eight weeks, the result confirmed that 1000 mg/day curcumin supplementation could decrease uric acid (*p* < 0.001) and serum lipids (*p* < 0.001) compared to the placebo group [87].

Coenzyme Q10 (CoQ10) is a natural compound that could positively affect the inflammatory status, the grade of hepatic steatosis, and liver enzymes' activity in patients with NAFLD. In a randomized, double-blind, placebo-controlled trial, 41 patients with NAFLD were divided into two groups and treated daily with 100 mg of CoQ10 capsule (intervention group) or placebo (placebo group) for three months. Results showed that the intervention group, compared to the control group, had a significant decrease in the grade of hepatic steatosis and serum levels of high-sensitivity C-reactive protein (*hs-CRP*), *AST*, gamma-glutamyl transpeptidase (*GGT*), and *TNF-α* (*p* < 0.05) [88].

In another study, Abidov et al. considered 151 volunteers (113 patients with NAFLD and 38 volunteers with normal liver fat). They treated them three times a day for 16 weeks with Xanthigen (300 mg brown seaweed extract containing 2.4 mg fucoxanthin + 300 mg pomegranate seed oil). The results showed that Xanthigen could significantly reduce body weight and body fat content in both groups and decrease waist circumference, liver fat content, and liver enzymes only in the NAFLD group. Hence, this natural drug has a promising function in obesity management [89].

Resveratrol is a natural compound whose therapeutic effect on NAFLD has been evaluated in many clinical trials. A previous study with forty patients showed that using this supplement (500 mg per day) for 12 weeks with adequate physical activity could significantly reduce steatosis grade, mass index, weight, waist circumference, and liver enzymes compared to the placebo group (medium-chain triglyceride) (*p* < 0.05). Resveratrol supplementation also significantly reduced inflammatory markers such as *IL-6*, *hs-CRP*, and *NF-κB* and hepatocellular apoptosis compared with the placebo group (*p* < 0.05). Furthermore, resveratrol supplementation with lifestyle modification was more effective than lifestyle modification alone [90].

Soy milk is another substance that affects the metabolic characteristics of NAFLD patients. The results of its eight-week consumption in 66 NAFLD patients showed that soy milk consumption (240 ml per day) could significantly diminish the level of serum insulin (*p*=0.04), homeostasis model assessment of insulin resistance (*p*=0.03), and blood pressure (*p*=0.04). It can also increase the quantitative insulin sensitivity check index (*QUICKI*) (*p*=0.04) compared to the control group [91]. The results of a randomized clinical trial conducted by Eslami et al. showed that 8-week administration of soy milk with a low-calorie diet in 70 patients could significantly reduce serum ALT and *hs-CRP* compared with the control group, which followed only a low-calorie diet (*p* < 0.05) [92]. Kani et al. conducted a parallel randomized clinical trial on 45 patients with grades 1 and 2 of NAFLD. They evaluated the efficacy of soy nut consumption on the serum leptin and inflammation level. Low-calorie diet, low-calorie low-carbohydrate diet, and low-calorie low-carbohydrate soy-containing diet (30 grams of soy nuts instead of 30 grams of red meat) were three groups, in which patients were randomized. After eight weeks, the results demonstrated a significant difference in reducing systolic and diastolic blood pressure, glycemic indices, fasting blood sugar (*FBS*), *hs-CRP*, and serum insulin level in the low-calorie low-carbohydrate soy-containing diet group compared to low-calorie or low-calorie low-carbohydrate diets. It has been found that these diets can move patients in grade 2 to grade 1, and the disease of some patients improved completely [93].

In a randomized controlled clinical trial, Gheflati et al. assessed the efficacy of purslane seeds in 54 individuals with NAFLD. Eight-week consumption of the purslane seeds (10 g/day) along with a low‐calorie diet significantly reduced serum concentrations of low‐density lipoprotein cholesterol (*LDL*), *FBS*, *QUICKI*, and total cholesterol, compared with the control group (only the low‐calorie diet) (*p* < 0.05) [94].

*Zataria multiflora* (ZM) is a thyme-like plant, a member of Lamiaceae family. A clinical trial evaluated the effect of *Zataria multiflora* in 85 patients with NAFLD. Patients were randomized to receive 700 mg plant powder (*n* = 45) or placebo (*n* = 40) twice daily for 12 weeks. Results revealed that *Zataria multiflora* could significantly reduce insulin resistance, insulin serum level, and blood pressure compared to the placebo group. However, there was no significant difference between *hs-CRP*, *TNF-α*, the grade of the fatty liver in ultrasonography, ALT, and other outcomes in the two groups [95].

Evaluation of the potential therapeutic effect of silymarin and vitamin E on liver tissue improvement in 36 NAFLD patients showed that daily intake of two tablets of silymarin (540.3 mg) and vitamin E (36 mg) with a hypocaloric diet for three months improved the noninvasive NAFLD index [96].

Previous studies demonstrated that ancient *Triticum turgidum* ssp. *turanicum (*Khorasan wheat), which is commercially known as Kamut, has a beneficial effect on human health. The results of comparing the treatment of Kamut to the control group (wheat products) in NAFLD patients with moderate liver steatosis showed a significant reduction of ALT, AST, alkaline phosphatase (ALP), and cholesterol in the Khorasan group (*p* < 0.05). Finally, Kamut could significantly improve the liver steatosis grade by reducing circulating proinflammatory *TNF-α*, *IL-8*, interferon-gamma (*IFNγ*), and the interleukin-1 receptor antagonist (*IL-1RA*) [97].

## 2. Modulation of miRNA Levels by Natural Products

Nowadays, plant secondary metabolites as multiple target compounds are widely used in disease treatment with complex pathogenesis. These compounds exert their therapeutic and pharmacologic effects by regulating the gene expression of critical signalling pathways [98, 99]. Another prominent mechanism of secondary metabolism is up- and downregulation of miRNA [100]. Various studies showed that the anticancer, anti-inflammatory, antihypercholesterolemic, antidiabetic, cardioprotective, and neuroprotective effects of alkaloids, flavonoids, coumarins, terpenes, iridoids, cardiac glycosides, and isothiocyanates are related to the regulation of miRNAs' expression [101–106]. For example, several investigations showed that berberine, an isoquinoline alkaloid isolated from different Berberidaceae and Ranunculaceae family species such as *Berberis vulgaris* and *Coptis chinensis*, regulated different miRNAs' expression [107–109]. Lu et al. reported that berberine suppressed microRNA-21 expression in the human colorectal cancer cell line (*HCT116*) at 100 *μ*M [110]. Also, osthole (20 mg/kg, twice a day, for six weeks) as prenylated coumarin showed its anti-Alzheimer's effects due to the increased miRNA-101a-3p expression in the cortex and hippocampus of mice [111]. Polyphenols are other phytochemicals that have regulation activity on miRNAs' expression [112]. Apigenin (4′,5,7-trihydroxyflavone) is a flavonoid, isolated from several genera including *Artemisia, Matricaria, Teucrium, Petroselinum, Apium,* and *Achillea* [113, 114], that has been shown to inhibit miRNA-103 expression at 40 mg/kg dose for 14 days intraperitoneally (i.p) in transgenic mice. The suppression of miRNA-103 ameliorated insulin sensitivity and glucose tolerance [115]. Downregulation of miRNA-29a has critical roles in various tumour and inflammatory diseases such as atherosclerosis, cholestasis, pediatric liver disease, and thoracic aneurysms [116]. Gracillin is a steroidal saponin that is mainly separated from *Dioscorea* spp. (Dioscoreaceae) [117]. This compound (10 mg/kg, i.p, 7 days) showed cardioprotective and anti-inflammatory effects due to an increase in the expression of miRNA-29a that had been suppressed by lipopolysaccharide (LPS, 10 mg/kg, i.p) in mice cardiomyocytes [118]. Antrocin [119], benzyl isothiocyanate [120], capsaicin [121], curcumin [122], quercetin [123], genistein [124], ginsenoside [125], emodin [126], oleuropein [127], resveratrol [128], and other secondary metabolites that could affect the regulation of miRNAs' expression and their natural sources are shown in Table 1.

### 2.1. Role of miRNA Signalling in Preventive and Therapeutic Potentials of Natural Products in NAFLD

**Figure 1:**
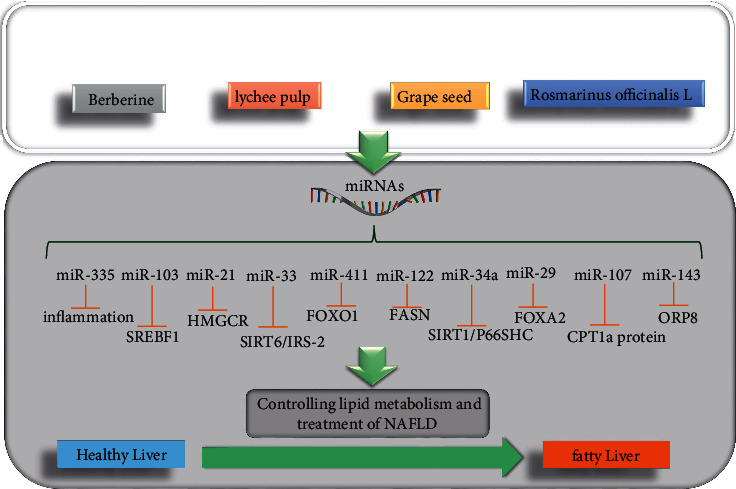
Natural products with preventive/therapeutic effects on NAFLD, acting via microRNAs.

Escalating evidence showed that natural products (the substances produced naturally by living organisms) possess protective or therapeutic effects against NAFLD (Figure 1) by regulating various microRNAs' expression. In a study, Adi et al. evaluated the identification of susceptibility genes and examined their diet behaviour. For this purpose, they studied the protective roles of a high-protein fish oil (HPO) diet on type 2 diabetes (T2D) and NAFLD in NONcNZO10 (NZ10) mice. Twelve mice were randomized to receive a control diet (CD). The other 12 mice were randomized to receive an HPO diet for 19 weeks, and microRNA expression profile changes and hepatic gene, steatosis, and blood chemistry were analyzed. The results confirmed that dietary protein and fish oil have protective effects against the development of T2D and NAFLD by downregulating miR-411 (>8-fold decrease by high glucose treatment of endothelial cells), miR-155 and miR-335 (>2-fold decrease by suppressing inflammation), miR-21 (>2-fold decrease as a marker of NAFLD by targeting HMGCR expression), miR-143 (>2-fold decrease by targeting the oxysterol-binding protein-related protein 8 (ORP8)), and miR-29a,b,c (>2-fold decrease by targeting FOXA2) [154]. Also, Wang et al. reported that the therapeutic potential of fish oil supplementation on cholesterol metabolic disorder and hepatic triglyceride is mediated through regulating the particular miRNAs' expression (rno-miR-33-5p and rno-miR-34a-5p) in Western-style diet-induced NAFLD rats [155].

Since recent studies have emphasized the miR-34a expression association with apoptosis in NAFLD, Shan et al. investigated the antiapoptotic effect of carnosic acid (CA), a phenolic compound extracted from the leaf of *Rosmarinus officinalis* (Lamiaceae), in fifty experimental rats that were randomly divided to receive CA or the high-fat diet (HFD) for ten weeks. The protective effect of CA against NAFLD was proved through the activation of SIRT1/p66shc by inhibiting miR-34a [58]. In another work, Yang et al. reported that the therapeutic potential of berberine on NAFLD is mediated through reducing liver uncoupling protein‐2 (UCP2) mRNA expression and the regulation of lipid metabolism. For this purpose, NAFLD rats were divided into the standard control group (regular diet with distilled water), the model control group (high-fat diet with distilled water), and the berberine group (high-fat diet with berberine solution). After 12 weeks, they found that, unlike the regular group that was devoid of protein expression, there was a significant increase in the model group.

Moreover, berberine significantly decreased the expression of UCP2 mRNA in comparison to the model group (*p* < 0.01) [156]. A phenolic-rich extract of lychee pulp (LPP) is useful for improving lipid metabolism in the liver by suppressing miR-33 and miR-122. This extract's hypolipidemic effects were studied by dividing mice into the HFD group or HFD combined with the LPP group. The result showed that the daily administration of the LPP for ten weeks could decrease the triglyceride, total serum cholesterol, fatty acid synthase mRNA, and corresponding protein expression levels [157]. Gracia et al. investigated the effect of resveratrol on miRNA-103-3p, miRNA-107-3p, and miRNA-122-5p expression in 16 rats, which were fed an obesogenic diet to induce liver steatosis. After 12 weeks of treatment, the results showed that resveratrol has protective effects against the development of liver fat by downregulating miR-103 (2.49-fold decrease), miR-107 (2.08-fold decrease by reducing carnitine palmitoyltransferase 1A (CPT1A) protein), and miR-122 (2.59-fold decrease by reducing FAS protein expression) [158]. Joven et al. demonstrated that the administration of plant-derived polyphenols for ten weeks was effective in hepatic metabolism, decreased liver steatosis, insulin resistance, and the expression of miR-103 and miR-107 in mice fed with the high-fat diet-induced fatty liver compared to the chow diet.

Moreover, polyphenols attenuated the expression of miR-122, which had not been altered with a fat-rich diet [159]. Grape seed proanthocyanidin extract (GSPE) effectively controls lipid metabolism by reducing liver fat regulators such as miR-33 and miR-122. The results showed that proanthocyanidin treatment decreased lipogenesis by repressing miR-12. Besides, it increased hepatic cholesterol efflux to procreate new HDL particles by suppressing miR-33 [160]. Baselga-Escudero and his colleagues studied the association of these miRNAs with lipemia. For this purpose, they analyzed these miRNAs in the livers of dyslipidemic cafeteria diet-fed rats and cafeteria diet-fed rats supplemented with proanthocyanidins and/or v-3 PUFAs. The results showed that, unlike the cafeteria diet, which showed an increasing effect, GSPE suppressed miR-33 and miR-122. However, SREBP2, the host gene of miR-33a, was significantly repressed by v-3 PUFAs but not by proanthocyanidins [161].

## 3. Conclusions

NAFLD is one of the most common metabolic diseases, which, in addition to its complications, is directly related to many other diseases such as cardiovascular problems, cancer, and kidney failure. Therefore, it is necessary to discover effective drugs due to widespread and critical fatty liver complications. Although various medications, such as thiazolidinediones, polyunsaturated fatty acids, and statins, have been proposed as invalid guidelines for NAFLD treatment, their effect on fatty liver treatment is not exact. On the contrary, identifying cellular mechanisms associated with NAFLD's occurrence and development, such as expression changes of related microRNA, including miR-34a, miR-155, miR-451, and miR-21, can be beneficial in finding effective treatments for this disease. The use of natural compounds for the treatment of NAFLD has long been considered. Various studies showed that the anticancer, anti-inflammatory, antihypercholesterolemic, antidiabetic, cardioprotective, and neuroprotective effects of alkaloids, flavonoids, coumarins, terpenes, iridoids, cardiac glycosides, and isothiocyanates are related to the regulation of miRNAs' expression. Various dietary fruits or medicinal herbal sources have received worldwide attention versus NAFLD development. So, natural compounds can cure various diseases, including NAFLD, by affecting the expression of microRNA. This study found that natural compounds such as polyphenols can play an essential role in improving and treating NAFLD by altering the expression of various microRNAs and NAFLD-related genes. However, extensive clinical studies are needed for their therapeutic approach in NAFLD patients.

## Figures and Tables

**Table 1:** Some secondary metabolites which show the pharmacological effects through the regulation of miRNAs.

Category structure	Secondary metabolite	miRNAs	Pharmacological results	Natural source^∗^	Ref.
Alkaloids	Berberine	↑miRNA-101	Blocking endometrial tumour growth and migration, *in vitro*, human endometrial cancer cell lines (AN3CA and HEC1A), 10, 30, and 100 *μ*M, 24 hours (h)	*Coptis chinensis* *=* *Coptidis Rhizoma* (Ranunculaceae), *Berberis vulgaris* (Berberidaceae)	[108]
		↓miRNA-122	Improving lipid hemostasis and hepatic gluconeogenesis, *in vivo*, diabetic mice, 40 mg/kg, 160 mg/kg, oral administration, 4 weeks	*Coptis chinensis* (Ranunculaceae), *Berberis vulgaris* (Berberidaceae)	[129]
		↑miRNA-203	Increasing the chemotherapy response of two cisplatin-resistant gastric cell lines 7901/DDP and BGC-823/DDP, *in vitro*, 10 *μ*M, 48 h	*Coptis chinensis* (Ranunculaceae), *Berberis vulgaris* (Berberidaceae)	[130]
	Rhynchophylline	↑miRNA-331-5p	Reducing ketamine dependence, *in vivo*, 60 mg/kg, i.p, 3 days	*Uncaria rhynchophylla* (Rubiaceae)	[131]
	Topsentin	↓miRNA-4485	Photoprotective effects, *in vitro*, human keratinocyte cell line (HaCaT), 2.5, 5, and 10 *μ*M, 6 h	*Spongosorites genitrix* (Halichondriidae)	[132]
	Tetrandrine	↓miRNA-155	Antidiabetic effects, *in vivo,* 100 mg/kg, i.p, mice, 48 h	*Stephania tetrandra* (Menispermaceae)	[133]
	Nicotine	↓miRNA-99b, ↓miRNA-192	Carcinogenicity, *in vitro*, non-small-cell lung cancer cell lines NCI-H460 and A549, 100 *μ*M, 48 h	*Nicotiana* spp. (Solanaceae)	[134]
	Camptothecin	↓miRNA-125b	Anticancer activity*, in vitro*, human cervical cancer (HeLa) and human immortalized myelogenous leukemia (K562) cell lines, 10 *μ*M, 48 h	*Camptotheca acuminata* (Nyssaceae)	[135]
	Palmatine	↑miRNA-200c	Suppressing breast cancer, *in vitro*, human breast cancer cell line (MCF-7), 10 *μ*M, 2 days	*Coptis chinensis* (Ranunculaceae)	[136]
	Vincristine	↓miRNA-34a	Anticancer activity against human retinoblastoma cell lines: HCT116 (CCL-247), WERI-Rb1 (HTB-169), *in vitro*, and Y79 (HTB-18), 2.5 nM, 48 h	*Catharanthus roseus* (Apocynaceae)	[137]

Coumarins	Osthole	↑miRNA-9	Anti-Alzheimer's activity, *in vitro*, neurons (from the cortex of neonatal mice), and human neuroblastoma cell line (SH-SY5Y), 50 *μ*M, 24 h	*Cnidium monnieri* (Apiaceae)	[138]
	Esculetin	↓miRNA-19b, ↑miRNA-30c	Cardioprotective activity, *in vitro*, human aortic endothelial cells (HAECs), 2.5 *μ*M, 2 h	*Artemisia capillaris,* (Asteraceae), *Citrus limonia* (Rutaceae)	[139]

Flavonoids	Apigenin	↓miRNA-122	Antihepatitis C virus, *in vitro*, human hepatoma cell line has an HCV replicon reporter construct (Huh7-Feo), 5 *μ*M, 5 days	*Matricaria chamomilla* (Asteraceae), *Apium graveolens* (Apiaceae)	[140]
	Chrysin	↑miRNA-9	Anticancer activity, *in vitro*, human gastric cell line (AGS), 35, 55, and 70 *μ*M, 24 h	*Passiflora* spp. (Passifloraceae)	[141]
	Genistein	↑miRNA-574-3p	Inhibiting proliferation on human prostate cancer cell line (PC3 and DU145), *in vitro*, 25 and 50 *μ*M, 24 h	*Glycine max* (Fabaceae)	[142]
	Resveratrol	↓miRNA-31	Treatment effects on ulcerative colitis, *in vivo*, 100 mg/kg, oral administration, 5 days	*Vitis* spp. (Vitaceae)	[128]
	Luteolin	↓miRNA-301-3p	Inhibiting proliferation, *in vitro*, human pancreatic cancer cell line, 25 and 50 *μ*M, 48 h	*Achillea millefolium* (Asteraceae)	[143]

Iridoids	Geniposide	↑miRNA-124a	Antirheumatoid arthritis activity, *in vitro*, human rheumatoid fibroblast-like synoviocyte line (MH7A), 50 *μ*M, 24 h	*Gardenia jasminoides* (Rubiaceae)	[144]
	Oleuropein	↓miRNA-519d	Increasing radiotherapy sensitivity, *in vitro*, human nasopharyngeal carcinoma cell lines (HNE1 and HONE1), 200 *μ*M, 24 h, *in vivo,* 1% w/v added to the mice drinking water, 7 days	*Olea europaea* (Oleaceae)	[127]
	Catalpol	↑miRNA-200	Anticancer activity, *in vitro* human ovarian cancer, (OVCAR-3), 50 and 100 *μ*g/ml, 48 h	*Rehmannia glutinosa* (Scrophulariaceae)	[145]

Isothiocyanates	Benzyl isothiocyanate	↑miRNA-99a	Anticancer activity, *in vitro,* human bladder cancer cell lines (5637 and T24), 10 and 20 *μ*M, 24 h	*Brassica oleracea* (Brassicaceae)	[120]
	Phenethyl isothiocyanate	↑miRNA-194	Anticancer activity, *in vitro,* human prostatic adenocarcinoma cell line (LNCaP and PC3), 2.5 *μ*M, 24 h	*Raphanus sativus* (brassicaceae)	[146]
	Allyl isothiocyanate	↓miRNA-155	Anti-inflammatory activity, mouse macrophage line (RAW264.7), 10 *μ*M, 24 h	*Brassica* spp. (Brassicaceae)	[147]

Quinones	Emodin	↑miRNA-34a	Suppressing liver tumour, *in vitro*, human liver cancer cell line (HepG2), 10 and 100 nM, 24 h, *in vivo*, hypodermic injection 1 and 10 mg/kg, 30 days	*Rheum palmatum, Polygonum cuspidatum*, *Polygonum multiflorum* (Polygonaceae)	[126]
	Shikonin	↑miRNA-140-5p	Reducing lung injury induced by sepsis, *in vitro*, mouse lung epithelial cells (MLE-12), 50 *μ*g/mL, 24 h, *in vivo*, specific pathogen-free (SPF) rat, 50.0 mg/kg, lingual vein injection, 6 h	*Lithospermum erythrorhizon* (Boraginaceae)	[148]

Saponins	Dioscin	↑let-7i	Nephroprotective activity, *in vitro,* standard rat kidney cell line (NRK-49F) and human kidney proximal tubular epithelial cell line (HK-2), 50, 100, and 200 ng/ml, 12 h, *in vivo,* Sprague Dawley (SD) rats (20, 40, and 80 mg/kg, i.p) and C57BL/6J mice (15, 30, and 60 mg/kg, i.p), 7 days	*Dioscorea* spp. (Dioscoreaceae)	[149]
	Ginsenoside Rg6	↑miRNA-146a	Reducing lung injury induced by sepsis, *in vitro*, bone-marrow-derived macrophage (BMDM) cell line, 20 *μ*M, 1 h, *in vivo,* mice, 20 mg/kg, i.p, (pretreatment for 2 h)	*Panax ginseng* (Araliaceae)	[125]
	Timosaponin A-III	↑miRNA-200c, ↑miRNA-141	Anticancer activity, *in vitro,* human breast adenocarcinoma cell lines (MDA-MB-231 and MCF7), 2, 4 *μ*M, 48 h	*Anemarrhena asphodeloides* (Asparagaceae)	[150]

Simple phenols	Capsaicin	↑miRNA-449a	Anticancer activity, androgen-sensitive human prostate adenocarcinoma cells (C4-2 and LNCaP), 100 *μ*M, 48 h	*Capsicum annuum* (Solanaceae)	[121]
	Curcumin	↑miRNA-34a	Anticancer activity, *in vitro,* human breast adenocarcinoma cell lines (MDA-MB-231 and MCF7), 30 *μ*M, 48 h	*Curcuma longa* (Zingiberaceae)	[122]
	Ferulic acid	↓miRNA-590	Improving spinal cord repair after injury, *in vitro*, neural stem cell (NSC), 10 *μ*M, 24 h	*Bambusa* spp. (Poaceae)	[151]

Terpenes	Paeoniflorin	↑miRNA-124	Anticancer activity, *in vitro*, human gastric cancer cell line (MGC-803), 20 *μ*M, 48 h	*Paeonia lactiflora* (Paeoniaceae)	[151]
	Triptolide	↑miRNA-137	Nephroprotective effects on diabetic rats, *in vitro*, human renal mesangial cell line (HRMC), 10 ng/ml, *in vivo*, 100 *μ*g/kg, oral administration, 12 weeks	*Tripterygium wilfordii* (Celastraceae)	[152]
	Betulinic acid	↑miRNA-27a	Anticancer activity, *in vitro*, human breast adenocarcinoma cell lines (MDA-MB-231), 2.5, 5, and 10 *μ*M, 24 h, *in vivo,* female athymic BALB/c nude mice, 20 mg/kg, oral administration, 25 days	*Betula* spp. (Betulaceae)	[153]
	Antrocin	↑let-7c	Anticancer activity, *in vitro*, non-small-cell lung cancer cell lines (H441), 5 *μ*M, 12 h	*Antrodia camphorata* (Polyporaceae)	[119]

^∗^The most important natural sources containing secondary metabolisms.
